# Synthesis of Plant-Inspired *O*‑Acetylated Hemicellulose Structures in the Yeast *Yarrowia lipolytica*

**DOI:** 10.1021/acssynbio.5c00595

**Published:** 2026-01-29

**Authors:** Marius Marcel Toni Karbach, Rajesh Kumar Natarajan, Nina Boots, Tim Niedzwetzki-Taubert, Markus Pauly, Vicente Ramírez

**Affiliations:** † Institute for Plant Cell Biology and Biotechnology, 9170 Heinrich Heine University Düsseldorf, 40225 Düsseldorf, Germany; ‡ Cluster of Excellence on Plant Sciences, Heinrich Heine University Düsseldorf, 40225 Düsseldorf, Germany

**Keywords:** hemicellulose, *O*-acetylation, microbial glycoengineering, Yarrowia lipolytica, plant cell wall, synthetic biology

## Abstract

Hemicelluloses are a group of plant cell wall polysaccharides characterized by their high structural complexity. These glycans are part of an intricate composite polymer network that contribute to the mechanical strength and flexibility of plant cell walls. Hemicellulose structural and functional diversity is further enhanced by the presence of chemical modifications, such as *O*-acetylation, altering the polysaccharide’s physicochemical properties and the overall functionality. Plant-derived hemicellulose glycans hold great promise for a range of biotechnological applications in a bioeconomy including biomaterials and pharmaceuticals. Synthetic biology approaches have the potential to produce hemicellulose polymers in microbial factories replicating the biosynthetic pathways observed in plants. In this study, we successfully reconstructed in the yeast *Yarrowia lipolytica* the biosynthesis of two hemicellulose backbone structures i.e., β-glucomannan (GM) and β-glucan, by the expression of glycosyltransferases of diverse plant origins. Oligosaccharide mass profiling combined with compositional and glycosidic linkage analysis confirmed the production of hemicellulose structures analogous to those found in the original plant systems. Furthermore, the additional expression of plant hemicellulose-specific *O*-acetyltransferases resulted in the biosynthesis of *O*-acetylated GM and *O*-acetylated glucan polymers, expanding the repertoire of hemicellulose structures produced in this yeast. These findings demonstrate the feasibility of generating not only compositionally diverse plant-like hemicellulose backbone polymers in microbial systems, but also more structurally complex *O*-acetylated variants beyond what is found in nature. The use of *Y. lipolytica* as a biofactory for designer glycans expands the potential of microbial glycoengineering and provides a platform for sustainable production of functionalized polysaccharides with tailored physicochemical properties optimized for specific biotechnological applications.

## Graphical abstract

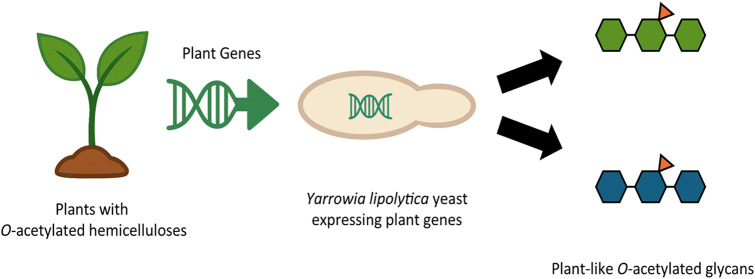

## Introduction

Plant hemicelluloses are a diverse group of polysaccharides residing in plant cell wall composites.^1^ The synthesis of hemicelluloses in plants is a complex, multistep process involving the coordinated activity of several glycosyltransferases (GTs).^2^ The resulting polysaccharide backbones can then be further decorated with additional sugar residues in defined patterns depending on the specific hemicellulose, plant cell type, and plant species.^2,3^ Noncarbohydrate modifications such as *O*-acetylation are also common in many hemicellulose types, conveying unique changes to the structure and shaping their properties including hydrophobicity and interactions with other wall polymers.^4–8^ Hemicellulose *O*-acetylation is catalyzed by acetyltransferases (AcT) from the Trichome Birefringence-Like (TBL) family, which transfer acetyl groups to specific glycans.^6,9^ In plants, GTs and AcTs work together in the Golgi apparatus to synthesize mature hemicellulose structures, which are then secreted via exocytosis and ultimately assembled into the plant wall.^6^

Significant progress has been made in identifying the key components involved in the synthesis and modification of most known hemicellulose types.^2^ This growing knowledge paves the way for innovative synthetic biology approaches, such as the production of plant-like polysaccharides in nonplant cell factories. Reconstructing the biosynthesis of these polymers in microbial hosts offers the potential to create designer glycan structures with tailored compositions and substitution patterns, allowing precise control over their physicochemical properties for specific applications.^10^ Plant-derived glycans currently have widespread applications as bioactive compounds in the food and feed industries, serving as sources of dietary fiber or modulators of gut microbiota composition.^11–13^ Similarly, the physiochemical properties of certain hemicellulose-like structures also make them attractive as gelling additives in cosmetics, pharmaceuticals and fracking/mining.^7,14^ Moreover, developing biofactories for customizable glycan structures offers exciting prospects in the growing field of sustainable biocomposites aimed at replacing plastics in packaging and construction, or use in soil amendments.^15^ Hemicellulose-derived glycans have also emerged as therapeutic agents in both human medicine and plant protection. Diverse hemicellulose structures have been shown to modulate immune responses, inhibit pathogen adhesion, or disrupt biofilm formation, making them valuable tools for disease prevention and treatment.^16^ Successful reconstruction of hemicellulose biosynthetic pathways in an orthogonal system relies on the selection of an appropriate host able to express a functional synthetic machinery. Yeast species are particularly well-suited for this purpose, providing higher-throughput, faster genetic engineering, and reduced wall glycan complexity compared to plants.^10^ Since many hemicellulose biosynthetic enzymes form multimeric complexes within the Golgi apparatus, the yeast endomembrane system—closely related to that of plants—facilitates likely proper protein folding, assembly, and localization. Like plants, yeasts produce activated NDP-sugars in the cytosol and have endogenous transporters capable of shuttling these substrates to the Golgi lumen for the use of the corresponding GTs.^17,18^ Furthermore, nucleotide sugar synthases of plant and microbial origins can be expressed in yeast to produce non-native sugar substrates such as UDP-xylose, UDP- glucuronic acid, or UDP-fucose, necessary for the synthesis of certain plant hemicelluloses.^19^ Production of basic hemicellulose structures in yeast has been achieved by engineering *Komagataella phaffii* cells (*Pichia pastoris*, Pichia) to express plant GTs. For example, Pichia cells expressing GT2 family members of the cellulose synthase-like A (CSLA) clade can produce glucomannan (GM) which accumulates in the yeast wall.^20^ Similar to GM found in plants, Pichia-produced GM consists of glucose (Glc) and mannose (Man) units β-1,4-linked in an unbranched backbone. The Man:Glc ratio of the polymer could also be modified by coexpressing specific accessory proteins, or swapping domains from CSLAs enzymes from different plant species, providing an example of the suitability of yeast systems to fine-tune the GM structure.^21^ Also in Pichia, the simultaneous expression of two GTs, namely CSLC4 and XXT1 (Xyloglucan Xylosyltransferase 1), resulted in the production of a β-1,4-linked glucan resembling the backbone of the plant hemicellulose xyloglucan (XyG).^21–23^

These pioneering synthetic biology studies have shown that yeasts can be used as biofactories to produce diverse hemicellulose backbone structures. These yeast-derived glycan scaffolds can now be further engineered to develop tailor-made glycans. Here, we report the successful reconstruction of the synthesis of *O*-acetylated GM (^Ac^GM) and *O*-acetylated glucan (^Ac^glucan) structures through the combinatorial expression of plant GTs and AcTs in the nonconventional yeast *Y. lipolytica* (Yarrowia), a model organism with unique metabolic capabilities which has previously been successfully engineered for various biomanufacturing purposes.^24,25^

## Results

### Selection of *Yarrowia lipolytica* as a Host to Produce *O*-Acetylated Hemicellulose Structures

Most yeast species endogenously produce UDP-glucose and GDP-mannose,^26^ precursors required for the synthesis of GM and glucan hemicellulose backbones. However, scarce information exists regarding the availability of acetyl precursors or even the presence of *O*-acetylated extracellular glycans in yeast, complicating efforts to reconstruct the *O*-acetylated hemicellulose biosynthetic pathways. The precise nature of these acetyl precursors in yeast, plants, or other eukaryotic systems remains elusive, although acetyl-CoA or derivative molecules have been proposed as likely candidates.^6,27^ *Y. lipolytica* is an oleaginous yeast with a high capacity for lipid production.^28,29^ As part of the enhanced fatty acid metabolism, Yarrowia produces large amounts of acyl-CoAs and derivatives,^30^ which could potentially also serve as substrates for hemicellulose *O*-acetylation. To assess its suitability as a host, we first determined whether Yarrowia cells contained native *O*-acetylated wall components. After isolation of the wall material, a weak base treatment leads to a quantitative release of acetate (Figure 1). For comparison, we included Pichia, *Saccharomyces cerevisiae* and maize wall material in the analysis. Yarrowia walls contain significant levels of acetate whereas Pichia and *S. cerevisiae* only contain negligible amounts. Although wall acetate levels in Yarrowia are lower than those found in plant tissues *i.e*., corn leaves, this result strongly suggests that unlike in Pichia and Saccharomyces, the extracellular matrix of Yarrowia is *O*-acetylated. Hence, Yarrowia contains an endogenous extracellular polysaccharide *O*-acetylation machinery and was thus selected as host to attempt the production of *O*-acetylated plant hemicellulose structures.

**Figure 1:**
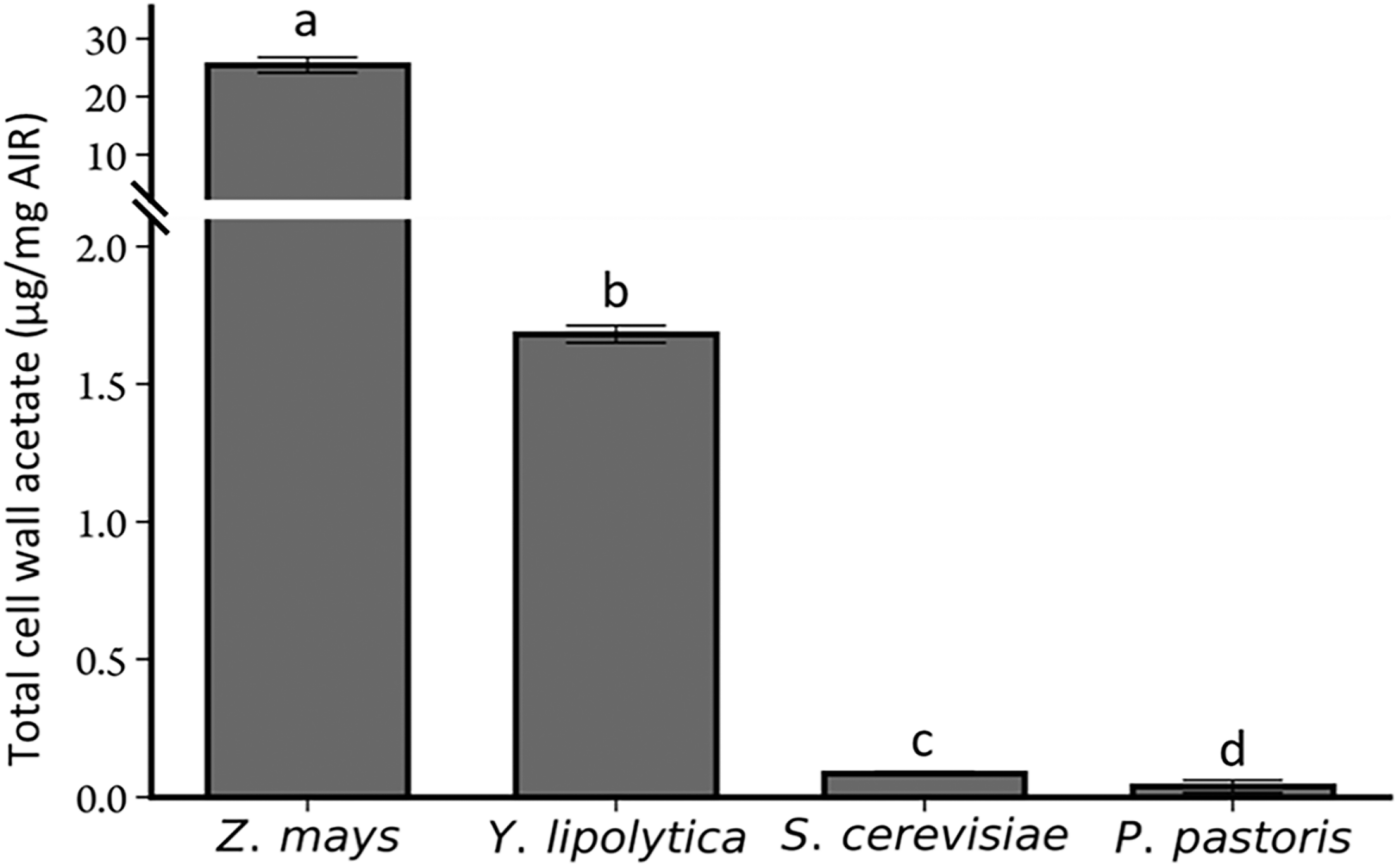
Acetate abundance in cell wall materials. Wall-bound acetate content in corn plants (*Zea mays*) and candidate orthogonal yeasts (*Y. lipolytica*, *S. cerevisiae*, and *P. pastoris*). Error bars represent the standard deviation, *n* = 3 biological replicates from individual cultures. Different letters indicate significant differences based on one-way ANOVA with post hoc Tukey HSD tests (*P* < 0.05).

### Production of *O*-Acetylated Glucomannan in *Y. lipolytica*

We explored the use of Yarrowia as a heterologous system to reconstruct the synthesis of ^Ac^GM. We selected GT and AcT genes from *Amorphophallus konjac*, since this plant accumulates large amounts of ^Ac^GM in its corm.^31^ A modular approach was used. First, Yarrowia cells were engineered to express the *Ak*CSLA3 GM synthase enzyme, sufficient to successfully produce GM in Pichia.^20^ A second module was engineered, consisting of the mannan-specific *Ak*TBL25 AcT.^32^ To minimize potential growth impacts on the yeast cells, the plant genes were placed under the control of an erythritol-inducible promoter (pEYK).^33^ Yarrowia strains expressing the *Ak*CSLA3 alone (*Yl* ^*AkCSLA3*^) or in combination with *Ak*TBL25 (*Yl* ^*AkCSLA3+AkTBL25*^) were generated. The potential production of GM and/or ^Ac^GM was monitored by oligosaccharide mass profiling (OLIMP).^34^ For this analysis, isolated wall material obtained from the different yeast strains was hydrolyzed by a specific endo-1,4-β-mannanase from *Cellvibrio japonicus* (*Cj*Man26A)^35^ and the resulting fragments were analyzed by matrix-assisted laser-desorption ionization time-of-flight (MALDI-TOF) mass spectrometry (Figure 2A). As a control, mannanase OLIMP on GM extracted from the plant *A. konjac* results in a characteristic ladder of *m*/*z* signals representing hexose oligosaccharides with degrees of polymerization (DP) ranging from 4 to 15 (689–2309 *m*/*z*). Additional signals corresponding to glycan-oligomers containing acetyl units (^*Ac*^GM oligosaccharides, +42 *m*/*z*) can be observed. Following a mild base treatment, these signals disappeared, consistent with the successful deacetylation of the oligosaccharides (Figure 2A).

**Figure 2:**
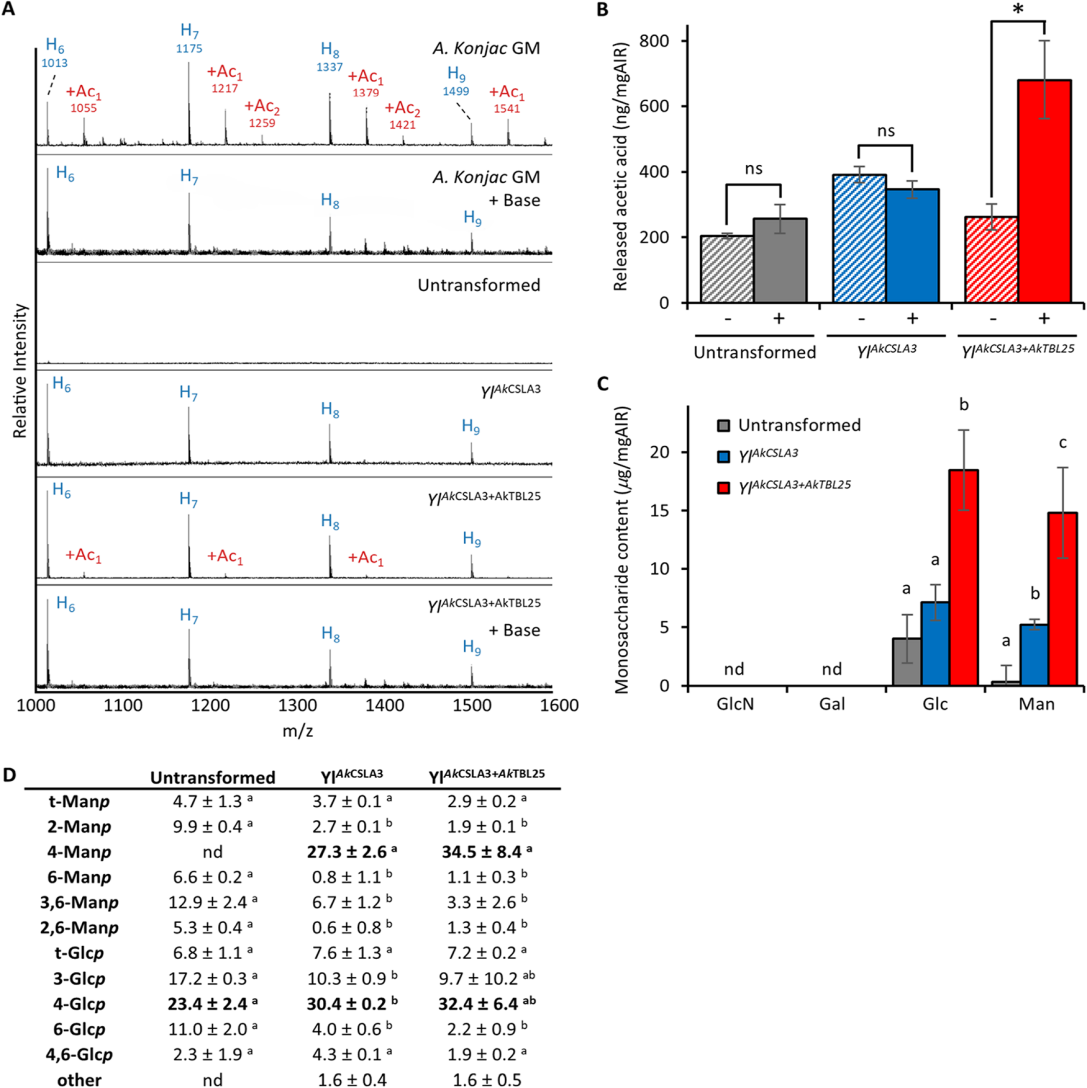
Characterization of Yl^AkCSLA3^ and Yl^AkCSLA3+AkTBL25^ strains. (A) Mannanase OLIMP of Yl^AkCSLA3^ and Yl^AkCSLA3+AkTBL25^. Wall material from the indicated strains was hydrolyzed with CjMan26A mannanase and the resulting oligosaccharides were analyzed by MALDI-TOF. Purified ^Ac^GM from the plant Amorphophallus konjac was used as control. Mass signals corresponding to oligosaccharides are annotated as [M + Na^+^] based on the mass-to-charge (*m*/*z*) ratio: H-hexose; Ac- *O*-acetyl-unit. Samples that showed putative *O*-acetylated signals were base treated removing acetylesters. (B) Acetic acid amounts detected after CjMan26A mannanase-digestion of wall material from Yl^AkCSLA3^, Yl^AkCSLA3+AkTBL25^ and untransformed strains (+) compared to a mock digestion performed without enzyme (−). Data shows the mean and standard deviation of three biological replicates from individual cultures. Symbols above bars indicate significant differences based on paired samples *t* test; ns = no significant difference, * = significant difference under *P* < 0.05. (C) Monosaccharide composition of mannanase-derived oligosaccharides isolated after SEC (SEC-oligosaccharides). Data shows the mean and standard deviation of three biological replicates from individual cultures for each monosaccharide type. Letters indicate significant differences based on one-way ANOVA with post hoc Tukey HSD tests (*P* < 0.05); nd = not detected. (D) Glycosidic linkage analysis of SEC-oligosaccharides. Linkages expected to be part of GM are highlighted in bold. Values correspond to the mean and standard deviation of three biological replicates from individual cultures. Different letters indicate significant differences based on one-way ANOVA with post hoc Tukey HSD tests (*P* < 0.05). nd: not detected; other: exact sugar derivative unknown.

While the same hydrolysis of untransformed Yarrowia walls did not yield any detectable oligosaccharides, *Cj*Man26A OLIMP on *Yl* ^*AkCSLA3*^ walls resulted in a ladder of hexose oligosaccharides with mass-to-charge (*m*/*z*) values consistent with deacetylated GM (Figure 2A). These results suggest that the sole expression of the *Ak*CSLA3 plant GT in Yarrowia leads to the production of a β-1,4-mannan polymer. In the case of *Yl* ^*AkCSLA3+AkTBL25*^, *Cj*Man26A OLIMP shows additional *m*/*z* signals matching those found in *A. konjac* ^Ac^GM. Base treatment of these oligosaccharides resulted in the disappearance of the +42 Da *m*/*z* signals (Figure 2A). To validate that the +42 Da signals indeed reflected acetyl modifications, the amount of acetate released after saponification of the mannanase hydrolysates was determined using a specific acetate enzyme assay. Mock-digested controls, in which the cell wall material was incubated under identical conditions without enzyme, were included to account for background solubilization or unspecific acetate release (Figure 2B). A significant increase in acetate was only found in *Yl* ^*AkCSLA3+AkTBL25*^ samples, confirming that mannanase-derived oligosaccharides were *O*-acetylated. These results indicate that the engineered Yarrowia strains simultaneously expressing *Ak*CSLA3 and *Ak*TBL25 are able to produce ^Ac^GM. For further structural characterization, the mannanase-released oligosaccharides from *Yl* ^*AkCSLA3*^ and *Yl* ^*AkCSLA3+AkTBL25*^ and the untransformed strains as a control were fractionated by size-exclusion chromatography (SEC). Following MALDI-TOF MS analysis on individual collected fractions (Figure S1), those containing (acetylated) oligosaccharides with an apparent molecular weight of 0.95–2.25 kDa were pooled for each strain resulting in an “SEC-oligosaccharide” sample. The SEC-oligosaccharides from the various yeast strains were subjected to acid hydrolysis, and the resulting monosaccharide compositions determined. Hydrolysis of untransformed Yarrowia total wall material yielded glucosamine, galactose, glucose (Glc), and mannose (Man), consistent with previous reports (10; Table S1). In contrast, the SEC-oligosaccharides from *Yl* ^AkCSLA3^ and *Yl* ^*AkCSLA3+AkTBL25*^ contained only significant amounts of glucose and mannose, as expected from GM production. Combining the monosaccharide composition and acetate measurements, ^Ac^GM produced by *Yl* ^*AkCSLA3+AkTBL2*^ carries roughly one acetyl group per ∼43.2 ± 11.1 Man/Glc units (Table S2). The Glc:Man ratio remained comparable between *Yl* ^*AkCSLA3*^ and *Yl* ^*AkCSLA3+AkTBL25*^ (1.2–1.4) (Figure 2C). However, the total amount of sugars detected in *Yl* ^*AkCSLA3+AkTBL25*^ was 2.5-fold higher than from *Yl* ^*AkCSLA3*^ indicating an increased abundance of GM. These findings suggest that while *O*-acetylation does not significantly alter GM composition, it might lead to a higher GM production and/or accumulation. One possibility is that *O*-acetyl moieties reduce aggregation of GM polysaccharide molecules rendering them more soluble, which might in turn increase the amount of ^Ac^GM accumulated in yeast cells. However, it cannot be ruled out that the mannanase hydrolysis is impacted by the presence of *O*-acetyl moieties due to steric hindrance, resulting in an apparent higher abundance of GM-derived oligosaccharides within the detected size ranges and hence higher levels of glucose and mannose in *Yl* ^*AkCSLA3+AkTBL25*^ -derived SEC-oligosaccharides. To further confirm the nature of the SEC-oligosaccharides, glycosidic linkage analysis was performed (Figure 2D). Consistent with the monosaccharide composition, only Man- and Glc-linkages were detected in *Yl* ^*AkCSLA3*^ and *Yl* ^*AkCSLA3+AkTBL25*^, with 4-Man*p* and 4-Glc*p* being the most abundant ones. The presence of small relative abundances of other linkages is indicative of residual contaminating carbohydrate fragments likely derived from glycogen, β1–3/β1–6 glucans and/or mannoproteins native to the yeast wall. These results indicate that the SEC-oligosaccharides are unbranched and composed of 4-linked Glc*p* and Man*p* residues confirming the successful reconstruction of the GM and ^Ac^GM biosynthetic pathways in Yarrowia.

### Production of an *O*-Acetylated Glucan in *Y. li* *polytica*

Next, we explored the potential of Yarrowia as a platform to produce glycan structures not naturally found in plants i.e., *O*-acetylated glucan (^Ac^glucan). The biosynthesis module consisted of two GTs—CSLC4 and XXT2 – both from Nasturtium (*Tropaeolum majus*), whose seeds contain large amounts of the storage polymer xyloglucan.^36^ Previous studies have shown that expressing both proteins increases the amount of synthesized xyloglucan glucan backbone compared to just CSLC4 alone.^21^ For the *O*-acetylation module, we selected Xyloglucan Backbone *O*-Acetyltransferase 1 (XyBAT1) from the grass *Brachypodium distachyon*. *Bd*XyBAT1 has been previously implicated in the *O*-acetylation of the xyloglucan glucan backbone and AcT activity on cellohexaose oligomers *in vitro* has been demonstrated.^37,38^ Similar to the GM-producing Yarrowia strains, the expression of those genes in Yarrowia was induced by erythritol and the yeast wall material was isolated and subjected to OLIMP analysis. To target glucan, the *Ba*Cel5 endo-1,4-β-glucanase from *Bacillus amyloliquefaciens* was used. *Ba*Cel5 OLIMP on β-glucan from barley flour resulted in a pattern of signals corresponding to hexooligosaccharides with a DP 8 to 16 (1337 – 2633 *m*/*z*). *Ba*Cel5 OLIMP on the untransformed strain did not show signals corresponding to oligosaccharide structures indicating the lack of endogenous 1,4-β-d glucan in Yarrowia walls (Figure 3A). In contrast, engineered yeast strains expressing *Tm*XXT2 and *Tm*CSLC4 show *m*/*z* profiles consistent with the presence of hexooligosaccharides, similar to those observed in β-glucan. *Ba*Cel5 OLIMP on *Yl* ^*TmXXT2+TmCSLC4+BdXyBAT1*^ strains revealed additional ion signals of +42 Da to each hexooligomer. These additional signals disappear after base treatment as expected from acetylester substituents (Figure 3A). Quantification of released acetate with an acetate specific enzyme assay further supported this interpretation as only endoglucanase hydrolysates from *Yl* ^*TmXXT2+TmCSLC4+BdXyBAT1*^ showed a significant increase compared to the mock treatment (Figure 3B). Together, these results indicate that the coexpression of *Tm*CSLC4 and *Tm*XXT2 genes in Yarrowia leads to the synthesis of a polysaccharide susceptible to endoglucanase hydrolysis, such as a 1,4-β-d glucan. Additional expression of *Bd*XyBAT1 results in *O*-acetylation of this polysaccharide. In order to confirm these findings, the oligosaccharides produced after endoglucanase digestion of *Yl* ^*TmXXT2+TmCSLC4*^ and *Yl* ^*TmXXT2+TmCSLC4+BdXyBAT1*^ walls were subjected to SEC, assessed by MALDI-TOF MS (Figure S1) and pooled as described for GM/^Ac^GM resulting again in an “SEC-oligosaccharide” fraction. Monosaccharide composition analysis of the SEC-oligosaccharides indicated large amounts of glucose, with minor amounts of mannose, galactose and glucosamine (Table S1). Together with the acetate measurements, ^Ac^glucan produced by *Yl* ^*TmXXT2+TmCSLC4+BdXyBAT1*^ would be carrying roughly one acetyl group per ∼ 9.7 ± 3.2 Glc units (Table S3). Similarly, linkage analysis showed an enrichment in 4-linked Glc*p* residues in *Yl* ^*TmXXT2+TmCSLC4*^ and *Yl* ^*TmXXT2+TmCSLC4+BdXyBAT1*^, which would indicate the production of a β-1,4-linked glucan (Figure 3C). However, both monosaccharide composition and linkage data showed the presence of additional sugars, likely resulting either from additional enzymatic activities of the endoglucanase used or partial solubilization of Yarrowia wall components during the reaction. In order to unambiguously determine the structure of the (acetylated) glycans produced, the SEC-oligosaccharides were subjected to reverse phase chromatography (RPC). MALDI-TOF analysis on the *Yl* ^*TmXXT2+TmCSLC4+BdXyBAT1*^ endoglucanase-derived products separated by RPC resulted in a separation of unacetylated and acetylated oligosaccharides (Figure 3D). RPC fractions eluting from 9 to 11 min contain primarily acetylated oligosaccharides, which were pooled resulting in an “RPC-oligosaccharide fraction”. Due to their low abundance as assessed by the MALDI-TOF relative intensity signals, compositional analyses of the RPC-oligosaccharides were performed by a more sensitive method - acetylated alditol derivatization followed by GC-MS analysis. The results indicated the acetylated RPC-oligosaccharides contained only glucose (Figure 3E). Taken together, the coexpression of the plant enzymes *Tm*XXT2 and *Tm*CSLC4 in Yarrowia leads to the production of a glucan polymer, which can be *O*-acetylated by *Bd*XyBAT1. Hence, the production of ^Ac^glucan, a glycan structure that is not naturally found *O*-acetylated in plants, could be demonstrated.

**Figure 3:**
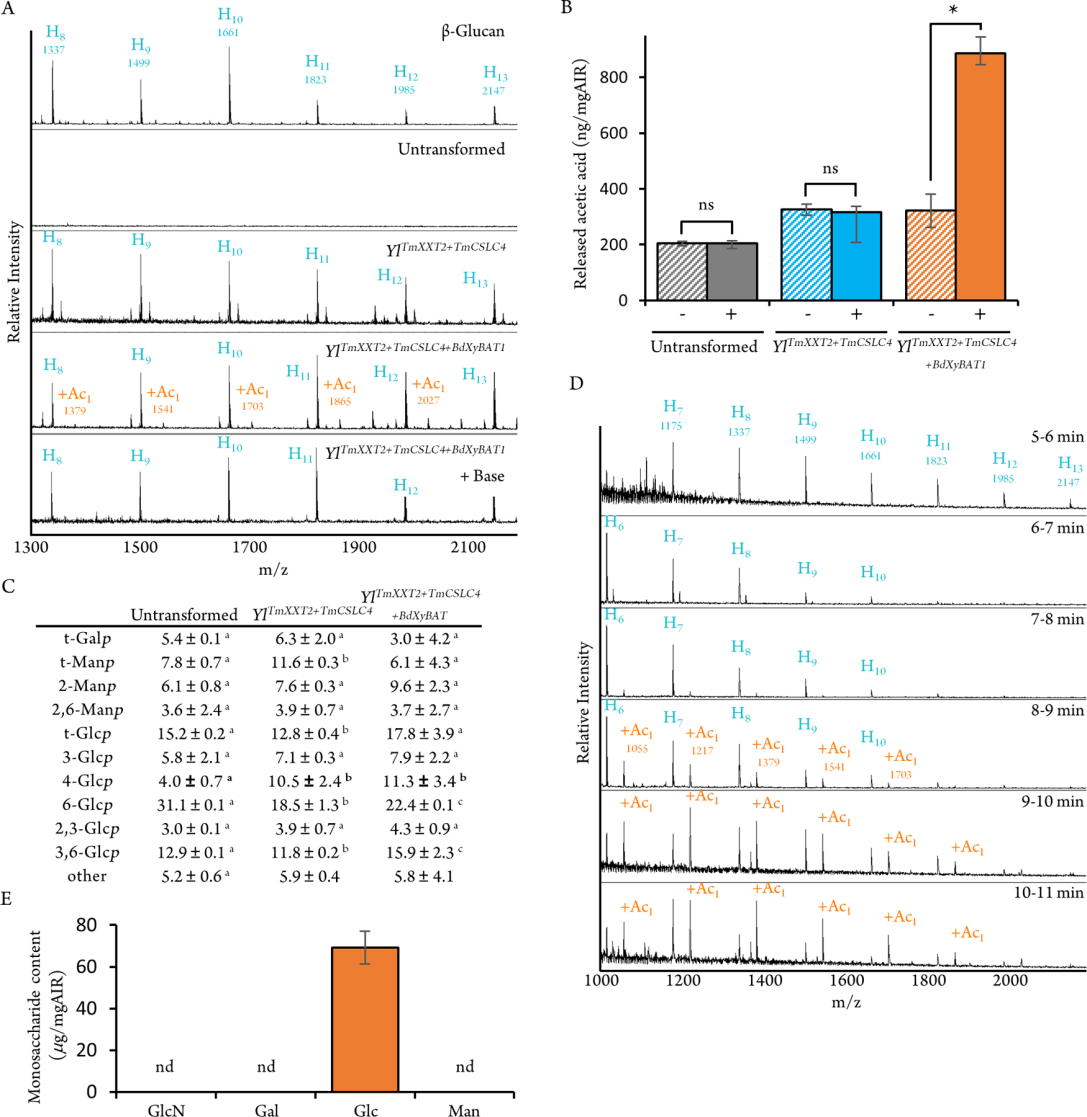
Characterization of Yl^TmXXT2+TmCSLC4^ and Yl^TmXXT2+TmCSLC4+BdXyBAT1^ strains. (A) Endoglucanase OLIMP of Yl^TmXXT2+TMCSLC4^ and Yl^TmXXT2+TMCSLC4+BdXyBAT1^. Wall material from the indicated strains was hydrolyzed with BaCel5 endoglucanase and analyzed by MALDI-TOF. β-Glucan from barley flour was used as a control. Ion signals corresponding to oligosaccharides are annotated as [M + Na^+^] based on the mass-to-charge (*m*/*z*) ratio: H-hexose; Ac- *O*-acetyl-unit. Samples that showed putative O-acetylated signals were base treated, removing acetylesters. (B) Amount of acetic acid detected after BaCel5 endoglucanase-digestion of wall material from Yl^TmXXT2+TMCSLC4^, Yl^TmXXT2+TMCSLC4+BdXyBAT1^ and untransformed strains (+) compared to a mock digestion performed without enzyme (−). Data shows the mean and standard deviation of three biological replicates from individual cultures. Symbols above bars indicate significant differences based on paired samples *t* test; ns = no significant difference, * = significant difference under *P* < 0.05. (C) Glycosidic linkage analysis of SEC-oligosaccharides. Linkages expected to be part of β-glucan are highlighted in bold. Values correspond to the mean and standard deviation of three biological replicates from individual cultures. Different letters indicate significant differences based on one-way ANOVA with post hoc Tukey HSD tests (*P* < 0.05). nd: not detected; other: exact sugar derivative unknown. (D) Preparation and analysis of an acetylated RPC-oligosaccharide fraction. The SEC-oligosaccharide fraction of Yl^TmXXT2+TmCSLC4+BdXyBAT1^ (Figure S1F) was subjected to RPC and the content of individual eluted fractions was assessed by MALDI-TOF MS. Shown are only fractions containing oligosaccharides (elution time: 5–11 min). (E) Monosaccharide composition of pooled RPC fractions (9–11 min) containing primarily acetylated oligosaccharides after acid hydrolysis. Data shows the mean and standard deviation of three biological replicates from individual cultures for each monosaccharide type. GlcN = glucosamine; Gal = galactose; Glc = glucose; Man = mannose; nd = not detected.

## Discussion

In this study we employed a synthetic biology approach to reconstruct the synthesis of plant-like hemicellulose structures in the yeast *Y. lipolytica*. Yarrowia strains were engineered to express various combinations of GTs and AcTs of plant origin to produce ^Ac^GM and ^Ac^glucan by hijacking endogenous pools of UDP-sugar and acetate donors. Given that our approach relies on the isolation and structural characterization of oligosaccharide fragments resulting from partial digestion with substrate-specific hydrolases, a precise quantification of the total hemicellulose structures produced is not feasible. However, the detailed compositional and glycosidic linkage analyses presented here provide clear and unambiguous evidence for the successful biosynthesis of the desired glycan structures in Yarrowia. Plant GT and AcT enzymes are often difficult to study biochemically due to their instability outside of their native membrane environment, as most are embedded in the Golgi via one or more transmembrane domains.^6^ Our results demonstrate that these membrane-bound enzymes remain functional when heterologously expressed in Yarrowia, efficiently utilizing native yeast nucleotide sugar substrates such as UDP-Glc and GDP-Man. These results pinpoint Yarrowia as a suitable orthogonal host for expressing functional GTs from diverse plant origins. Likewise, the production of *O*-acetylated structures indicates that Yarrowia’s endogenous acetyl substrates can be utilized by plant AcTs suggesting a conserved glycan *O*-acetylation precursor metabolism in plants and yeast. Our synthetic platform thus provides a flexible and tractable system to gain a mechanistic understanding of the actions of GTs and accessory proteins with unknown or ambiguous substrate specificities. It offers a practical alternative to labor-intensive *in vitro* assays or *in planta* studies, which are often complicated by genetic redundancy or severe developmental phenotypes associated with hemicellulose hypoacetylation.^10,39^

Besides serving as a platform for orthogonal host research approaches, mastering the application of enzyme-derived *O*-acetylation can offer unique advantages compared to more traditional, organic chemistry-based *O*-acetylation procedures. Conventional chemical procedures use acetylation reagents such as acetic anhydride to acetylate target molecules and can be successfully applied on large scales. However, acetic anhydride and similar reagents do not discriminate between nucleophilic target sites within a molecule and thus usually lead to the acetylation of several or all possible acetylation targets (such as OH-groups in sugars). If more selective acetylation is desired for a procedure, it requires labor-intensive adjustments to protect undesired target sites from acetylation.^40^ In contrast, enzymatic acetylation as utilized here, usually targets very specific polymers and target sites. For example, the *Arabidopsis thaliana* xylan *O*-acetyltransferase XOAT1 – a member of the TBL-family of proteins – exclusively targets the *O*-2 position of the xylosyl-residues in xylans.^41^ We also observe this in our results: Figures 2A and 3A show that our plant glycan oligos are exclusively monoacetylated. For natural enzyme targets, our enzymatic approach may thus provide a viable alternative to classic chemical procedures by harnessing the naturally specific acetylation patterns of enzymes.

Notably, the procedure is not only limited to natural substrates. While ^Ac^GM is a natural component of plant cell walls, ^Ac^glucan is not. The ability to produce such glycan structures bearing non-natural *O*-acetylation patterns underscores the broader potential of this platform, not only to mimic plant structures, but also for designing and producing novel glycan architectures beyond those found in nature. Such designer glycans could serve as valuable tools for developing tailor-made biomaterials with customizable functionalities and properties, opening new possibilities for applications in feed, food, and pharmaceutical biotechnology.

## Methods

### Constructs, Strains and Media

All plasmids and strains used in this study are listed in Tables S4 and S5. Plasmid backbones for expression in *Y. lipolytica* were obtained from the EasyCloneYALI collection kit.^42^ Genes of interest were amplified by PCR using Phusion High-Fidelity DNA Polymerase (Thermo Fisher Scientific) and cloned into the plasmid backbones using the Gibson Assembly Cloning kit (NEB). Plant gene sequences were obtained from *A. thaliana* Columbia-0, *A. konjac*, *T. majus*, and *B. distachyon*. The available GenBank accession numbers are HQ833588 (*Ak*CSLA3 ^43^), MH663995 (*Ak*TBL25 ^32,43^ ), and XM_003569420 (*Bd*XyBAT^44^). No database entry is available for the *T. majus* genes, but their sequences were sourced from^22^ for *Tm*CSLC4 and from^36^ for *Tm*XXT2. The pEYK promoter sequence for erythritol-induced gene expression was obtained from unmodified *Y. lipolytica*.^33^ All the yeast strains in this study were derived from *Y. lipolytica* Po1d from Barth and Gaillardin.^45^ DNA constructs were transformed into *Y. lipolytica* strains using a lithium-acetate transformation method as described before.^42^ *Escherichia coli* was grown in lysogeny broth (LB) or on LB agar plates supplemented with 10 g/L agar. For selection, 100 mg/L ampicillin were added to the media or plates. *Y. lipolytica* strains were grown in yeast peptone dextrose medium (YPD) or on YPD supplemented with 10 g/L agar for plate cultures. When applicable, media or plates were supplemented with 250 mg/L nourseothricin for antibiotic selection. For erythritol induction of gene expression in *Y. li* *polytica*, 25 mL precultures were grown in 125 mL baffled flasks containing yeast nitrogen base media (YNB, Sigma-Aldrich) supplemented with 5.3 g/L ammonium chloride, 0.1 g/L leucine and 0.1 g/L uracil and 10 g/L glucose for 3 days at 30 °C and 225 rpm. After 3 days, YNB medium was removed through 2 min of centrifugation at 3000*g* and the cells were washed and resuspended in an equal volume of YNB medium supplemented with 20 g/L erythritol instead of glucose as carbon source. The induction was applied for 3 days with the YNB + erythritol medium being refreshed daily. All following data was obtained from three replicates of independent inoculums of three independent transformants of each strain.

### Cell Wall Polymer Isolation

The induced *Y. lipolytica* cells were harvested by centrifugation. Alcohol insoluble residue (AIR) preparation was conducted to extract cell wall material. Cells were mechanically ground up with 0.1 mm glass beads (Sigma-Aldrich) in 70% aqueous ethanol for 10 min in a mixer mill MM400 (Retsch) at 30 Hz. After collecting the material through centrifugation and removing the supernatant, the pellet was washed first with chloroform:methanol (1:1 v/v) and then with acetone, each time grinding the material again for 10 min at 30 Hz. After drying, the AIR material was destarched. The AIR pellet was resuspended in 0.1 M pH 5 citrate buffer, broken up in the mixer mill with a steel ball at 30 Hz for 5 min, and digested with 15 U α-amylase and 20 U of pullulanase, supplemented with 0.2 μg/mL of sodiumazide at 37 °C and 220 rpm for 16 h. Insoluble destarched wall material was harvested by centrifugation and washed once with water and twice with acetone. After drying, the destarched AIR pellet was further treated with zymolyase. The AIR material was milled and resuspended in a reaction mixture containing 75 U zymolyase in water supplemented with 0.2 μg/mL sodiumazide. The reaction was incubated at 37 °C and 225 rpm for 16 h. The insoluble material was washed twice with 70% ethanol and once with acetone. Once dried, wall material was resuspended in water to a final concentration of 20 mg/mL and stored frozen until further use.

### Oligosaccharide Mass Profiling

OLIMP was performed as previously described.^34^ Cell wall material (20 mg/mL) was mixed with 1 U/ml of *endo*-1,4-β-mannanase from *Cellvibrio japonicus* (E-BMACJ, Megazyme) or *endo*-1,4-β-d-glucanase from *Bacillus amyloliquefaciens* (E-CELBA, Megazyme). After 1.5 h incubation at 40 °C, the supernatant was spotted onto a mass spectrometer target plate on dried 10 mg/mL 2,5-dihydroxybenzoic acid matrix supplemented with 10 mmol/mL sodium chloride and analyzed on a RapifleX matrix-assisted laser-desorption ionization time-of-flight mass spectrometer (MALDI) (Bruker Daltonic) in positive reflectron mode with an acceleration voltage of 20 kV. When indicated, OLIMP samples were base-treated to remove acetylation-modifications from oligosaccharides for visualization during MALDI-TOF MS. For this, mannanase or glucanase hydrolysates were mixed with an equal volume of 0.5 M ammonia solution and incubated at 25 °C for 1 h while rotating at 1000 rpm. After neutralization with formic acid, solutions were dried using a Concentrator plus SpeedVac (Eppendorf). Samples were then resuspended in one volume of water and analyzed by MALDI-TOF MS as described above.

### *O*-Acetylation Quantification

The acetate contents in plant, yeast and OLIMP-derived samples were determined as in.^46^ Briefly, samples were treated with 0.5 M sodium hydoxide for 1 h at 25 °C, agitated at 1000 rpm. The solution was neutralized with half the original volume of 1 M HCl and the acetic acid released in the supernatant was spectrophotometrically determined by the Acetic Acid Assay Kit (K-ACET, Megazyme).

### Size-Exclusion Chromatography

The supernatants of the OLIMP digest were separated using an NGC Scout 10 chromatography system (Bio-Rad), equipped with a Superdex Peptide Column 10/300 GL (Cytiva). The eluent (water) was assessed by a RID-20A refractive index detector (Shimadzu). Fractions were collected for mass spectrometrical analyses by an NGC Fraction Collector (Bio-Rad). Fractions containing oligosaccharides were pooled (SEC-oligosaccharides) and dried with a Concentrator plus SpeedVac (Eppendorf).

### Monosaccharide Composition Analysis

Monosaccharide composition analysis was performed using high-performance anion-exchange chromatography (HPAEC) in an Azura HPAEC system (Knauer) equipped with a Dionex CarboPac Pa20 column (Thermo Fisher Scientific) and a pulsed amperometric detector (PAD) according to protocols described previously.^21^ The resulting peaks in the chromatogram were assigned based on their retention times to sugar standards.

### Glycosidic Linkage and Alditol Acetate Analysis

Experimental procedures for methylation, hydrolysis, reduction and acetylation derivatization were performed as previously described.^20^ For monosaccharide composition analysis via alditol acetate derivatization, the methylation steps were omitted as previously described.^47^ The GC/MS device used was a 7890B GC system (Agilent) connected to a 5977A series Quadrupole MS system (Agilent). GC separation was conducted using a SP2380 capillary column (Supelco). Each carbohydrate peak was annotated according to its retention time and ion fragmentation spectrum in comparison to a PMAA database based on standards.^48^

### Reversed Phase Chromatography (RPC)

SEC-oligosaccharides were further enriched using reversed phase chromatography (RPC) according to previously described methods.^49^ For this purpose, the dried SEC-oligosaccharide fraction was resuspended in 6% methanol and subjected to an Azura HPAEC system (Knauer) equipped with a Vydac 238TP 5 μL C18 (Avantor) column. The device was connected to an evaporative light scattering detector (ELSD, model, company) to detect eluting compounds. The methanol eluent was applied at a flow rate of 0.5 mL/min at a gradient, ramping up from an initial 6% methanol to 50% methanol at 70 min, after which the gradient was reversed back to 6% methanol within 5 min. Fractions containing the desired oligosaccharides were pooled (RPC-oligosaccharides).

## Supplementary Material



## Notes

Detailed GMO information and additional experimental results, including MALDI spectra of SEC-fractions, and further monosaccharide composition data of other experimental steps (PDF)

## §

School of Biological Sciences, The University of Western Australia, Perth, 6009, Western Australia, Australia
