# Supplementary material for: Synthesis of Plant-Inspired O‑Acetylated Hemicellulose Structures in the Yeast Yarrowia lipolytica
Source: ACS Synth Biol. 2026 Jan 29;15(2):490–8. doi: 10.1021/acssynbio.5c00595 (PMC12930491; doi:10.1021/acssynbio.5c00595)
Supplement: Supplementary file 1 [file sb5c00595_si_001.pdf]

# Supporting Information

## Synthesis of plant-inspired *O*-acetylated hemicellulose structures in the yeast *Yarrowia lipolytica*

Marius Marcel Toni Karbach[1], Rajesh Kumar Natarajan[1,2], Nina Boots[1], Tim Niedzwetzki-Taubert[1], Markus Pauly[1,3] and Vicente Ramírez[1]*

[1] Institute for Plant Cell Biology and Biotechnology, Heinrich Heine University Düsseldorf, 40225, Düsseldorf, Germany.

[2] Current address: Batley Lab, Plant Genomics for Resilient Crops and Sustainable Agriculture, The University of Western Australia, WA6009, Perth, Australia

[3] Cluster of Excellence on Plant Sciences, Heinrich Heine University Düsseldorf, 40225, Düsseldorf, Germany

* Corresponding author: ramirezg@hhu.de

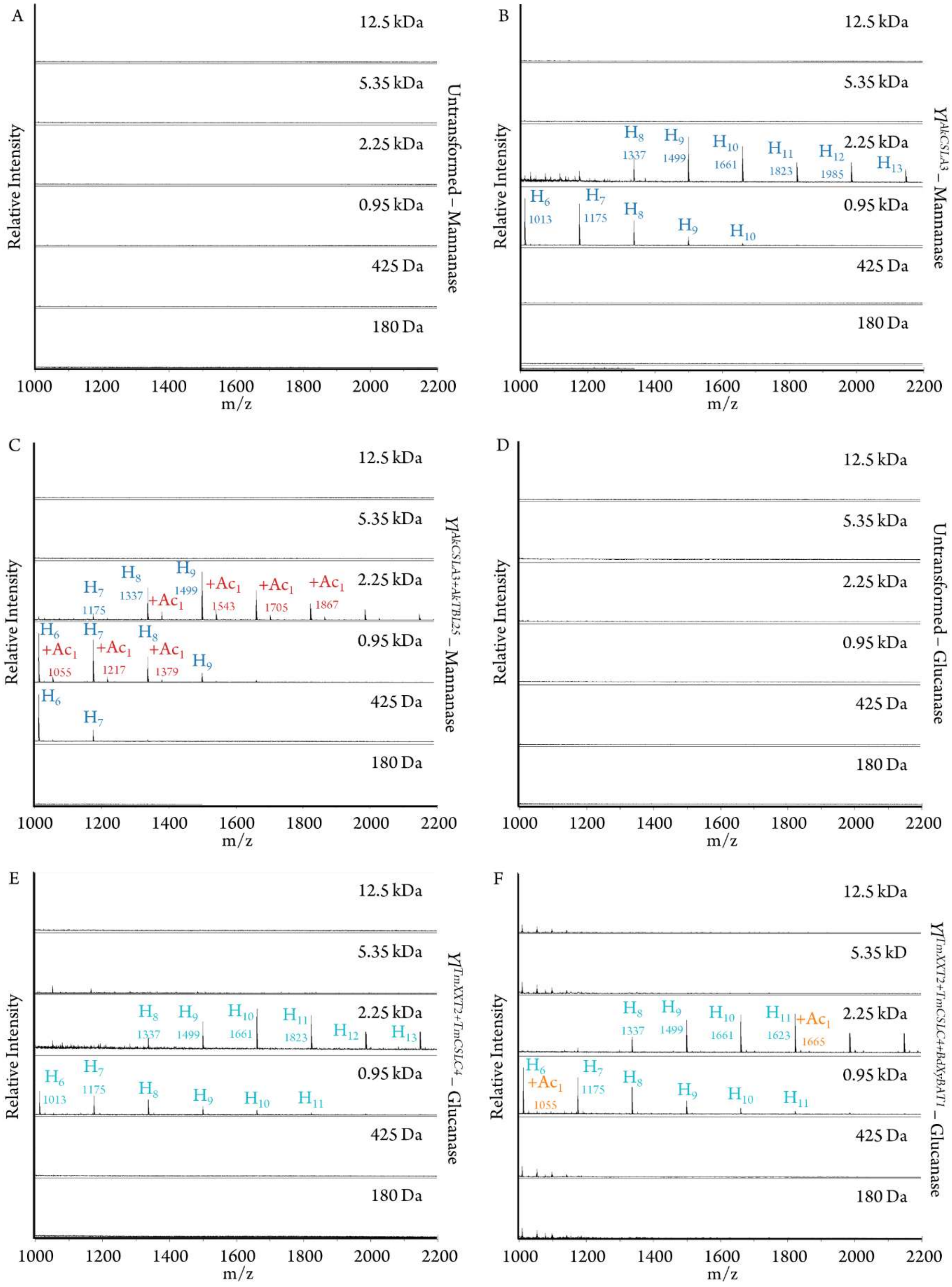


**Figure S1. MALDI-TOF spectra of SEC-separated fractions.** (A) MALDI-TOF spectra of SEC-fractions of untransformed strain after *Cj*Man26A-digestion. (B) MALDI-TOF spectra of SEC-fractions of $Yl^{AkCSLA3}$-strain after *Cj*Man26A-digestion. (C) MALDI-TOF spectra of SEC-fractions of $Yl^{AkCSLA3+AkTBL25}$ strain after *Cj*Man26A-digestion. (D) MALDI-TOF spectra of SEC-fractions of untransformed strain after *Ba*Cel5-digestion. (E) MALDI-TOF spectra of SEC-fractions of $Yl^{TmXXT2+TmCSLC4}$ strain after *Ba*Cel5-digestion. (F) MALDI-TOF spectra of SEC-fractions of $Yl^{TmXXT2+TmCSLC4+BdXyBAT1}$ strain after *Ba*Cel5-digestion.

**Table S1. Complete table of monosaccharide composition analysis of *Y. lipolytica* strains.** Data was obtained from untreated AIR material before any OLIMP experiments (row 1), the undigested residue after OLIMP treatment with *Cj*Man26A or *Ba*Cel5 (row 2), the supernatant containing the total of solubilized sugars after OLIMP treatment with *Cj*Man26A or *Ba*Cel5 (row 3), and the SEC-oligosaccharides containing material of SEC-fractions corresponding to 0.95 kDa and 2.25 kDa from Fig. S1 for all modified and untransformed strains. Mean + standard deviation of three biological replicates from individual cultures are depicted for each monosaccharide type. For each monosaccharide or sample, different letters indicate significant differences based on one-way ANOVA with post hoc Tukey HSD tests ($P < 0.05$); nd = not detected.

**Untreated AIR Material**

| Sugars (µg) | Untransformed | $Yl^{AkCSLA3}$ | $Yl^{AkCSLA3+AkTBL25}$ | $Yl^{TmXXT2+TmCSLC4}$ | $Yl^{TmXXT2+TmCSLC4+BdXyBAT1}$ |
|---|---|---|---|---|---|
| Glucosamine | 74.0 ± 24.1 $^{a}$ | 59.2 ± 7.8 $^{a}$ | 70.6 ± 17.4 $^{a}$ | 65.5 ± 1.5 $^{a}$ | 70.5 ± 0.7 $^{a}$ |
| Galactose | 68.8 ± 7.2 $^{a}$ | 61.7 ± 2.0 $^{a}$ | 73.1 ± 14.5 $^{a}$ | 49.4 ± 0.4 $^{a}$ | 50.6 ± 1.7 $^{a}$ |
| Glucose | 376.7 ± 22.7 $^{a}$ | 375.5 ± 26.2 $^{a}$ | 411.2 ± 14.4 $^{a}$ | 267.9 ± 6.0 $^{b}$ | 243.2 ± 32.8 $^{b}$ |
| Mannose | 232.0 ± 15.0 $^{a}$ | 235.0 ± 6.7 $^{a}$ | 291.4 ± 35.8 $^{b}$ | 227.2 ± 2.1 $^{a}$ | 230.6 ± 5.8 $^{a}$ |

**Undigested Residue**

| | Mannanase | | | Glucanase | | |
|---|---|---|---|---|---|---|
| Sugars (µg) | Untransformed | $Yl^{AkCSLA3}$ | $Yl^{AkCSLA3+AkTBL25}$ | $Yl^{TmXXT2+TmCSLC4}$ | $Yl^{TmXXT2+TmCSLC4+BdXyBAT1}$ | Untransformed |
| Glucosamine | 49.9 ± 18.4 $^{a}$ | 46.1 ± 7.6 $^{a}$ | 54.4 ± 14.2 $^{a}$ | 114.8 ± 5.9 $^{a}$ | 129.1 ± 13.5 $^{a}$ | 46.5 ± 33 $^{b}$ |
| Galactose | 17.4 ± 6.4 $^{a}$ | 16.1 ± 2.3 $^{a}$ | 21.2 ± 3.9 $^{a}$ | 31.6 ± 2.8 $^{a}$ | 29.7 ± 5.3 $^{a}$ | 9.1 ± 8.9 $^{a}$ |
| Glucose | 253.3 ± 40.3 $^{a}$ | 256.4 ± 32.6 $^{a}$ | 272.6 ± 22.4 $^{a}$ | 261.5 ± 17.2 $^{a}$ | 215.1 ± 87.6 $^{a}$ | 147.9 ± 105.4 $^{a}$ |
| Mannose | 48.7 ± 11.2 $^{a}$ | 51.8 ± 9.1 $^{a}$ | 64.8 ± 5.9 $^{a}$ | 95.3 ± 11.1 $^{a}$ | 81.6 ± 16.7 $^{a}$ | 56.7 ± 32.5 $^{a}$ |

**Supernatant**

| | Mannanase | | | Glucanase | | |
|---|---|---|---|---|---|---|
| Sugars (µg) | Untransformed | $Yl^{AkCSLA3}$ | $Yl^{AkCSLA3+AkTBL25}$ | $Yl^{TmXXT2+TmCSLC4}$ | $Yl^{TmXXT2+TmCSLC4+BdXyBAT1}$ | Untransformed |
| Glucosamine | 8.0 ± 2.9 $^{a}$ | nd | nd | 5.5 ± 1.2 $^{a}$ | 8.7 ± 0.5 $^{a}$ | 5.8 ± 0.1 $^{a}$ |
| Galactose | 40.1 ± 14.8 $^{a}$ | 38.1 ± 0.5 $^{a}$ | 41 ± 10.1 $^{a}$ | 32.9 ± 3.1 $^{a}$ | 28.6 ± 2.0 $^{a}$ | 11.4 ± 1.6 $^{b}$ |
| Glucose | 179.5 ± 23.1 $^{a}$ | 173.9 ± 2.2 $^{a}$ | 182 ± 0.3 $^{a}$ | 94.7 ± 11.3 $^{a}$ | 106.1 ± 12.8 $^{a}$ | 76.8 ± 1.7 $^{b}$ |
| Mannose | 178.2 ± 65.0 $^{a}$ | 216.6 ± 3.9 $^{a}$ | 263.4 ± 49.6 $^{a}$ | 73.2 ± 4.4 $^{a}$ | 71.3 ± 2.6 $^{a}$ | 54.8 ± 3.9 $^{b}$ |

**SEC-Oligosaccharides**

| | Mannanase | | | Glucanase | | |
|---|---|---|---|---|---|---|
| Sugars (µg) | Untransformed | $Yl^{AkCSLA3}$ | $Yl^{AkCSLA3+AkTBL25}$ | $Yl^{TmXXT2+TmCSLC4}$ | $Yl^{TmXXT2+TmCSLC4+BdXyBAT1}$ | Untransformed |
| Glucosamine | nd | nd | nd | 5.4 ± 1.2 $^{a}$ | 13.8 ± 6.1 $^{a}$ | 2.1 ± 0.1 $^{b}$ |
| Galactose | nd | nd | nd | 5.6 ± 0.3 $^{a}$ | 3.2 ± 0.4 $^{b}$ | 3.4 ± 0.9 $^{b}$ |
| Glucose | 4.0 ± 2.1 $^{a}$ | 7.1 ± 1.5 $^{a}$ | 18.4 ± 3.4 $^{b}$ | 159.5 ± 26.9 $^{a}$ | 227.2 ± 18.3 $^{a}$ | 215.4 ± 47.6 $^{a}$ |
| Mannose | 0.3 ± 1.4 $^{a}$ | 5.2 ± 0.4 $^{b}$ | 14.8 ± 3.9 $^{c}$ | 18.6 ± 0.5 $^{a}$ | 23.6 ± 2.7 $^{b}$ | 11.0 ± 1.6 $^{c}$ |

Table S2 Released acetic acid to glucose and mannose ratios of *Cj*Man26A-mannanase digested wall material from $Yl^{AkCSLA3+AkTBL25}$

| Sample | Glucose + Mannose Content (nmol) | Acetc Acid Content (nmol) | Released Acetic Acid/Glucose+Mannose ratio (mol/mol) | Monosaccharides per acetyl group |
|---|---|---|---|---|
| **Mock Digestion** | 82.7 ± 13.1 | 7.5 ± 1.4 | | |
| **Mannanase Digestion** | 492.5 ± 12.8 | 15.8 ± 2.8 | | |
| **Difference** | 409.8 ± 27.9 | 9.7 ± 1.9 | 0.02396 ± 0.00617 | 43.2 ± 11.1 |

Table S3. Acetic acid to glucose ratios of *Ba*Cel5-glucanase digested wall material from $Yl^{TmXXT2+TmCSLC4+BdXyBAT1}$

| Sample | Glucose Content (nmol) | Acetc Acid Content (nmol) | Released Acetic Acid/Glucose ratio (mol/mol) | Monosaccharides per acetyl group |
|---|---|---|---|---|
| **Mock Digestion** | 91.9 ± 22.7 | 7.5 ± 1.4 | | |
| **Mannanase Digestion** | 215.6 ± 3.1 | 20.6 ± 0.9 | | |
| **Difference** | 123.8 ± 19.6 | 13.1 ± 2.3 | 0.10861 ± 0.03570 | 9.7 ± 3.2 |

Table S4. Plasmids used in this work.

| Plasmid | Parental Plasmid | Added Cassette | Application | Origin |
|---|---|---|---|---|
| pCfB4780 | / | / | Origin Vector | EasyCloneYALI kit (Holkenbrink et al., 2018) |
| pCfB4780+*Ak*CSLA3 | pCfB4780 | pEYK, *Ak*CSLA3, Tlip | Expression of *Ak*CSLA3 | This study; Gibson Assembly |
| pCfB4780+*Ak*CSLA3+*Ak*TBL25 | pCfB4780 | pEYK, *Ak*CSLA3, *Ak*TBL25, Tlip | Expression of *Ak*XCSLA3+*Ak*TBL25 | This study; Gibson Assembly |
| pCfB4780+*Tm*XXT2+*Tm*CSLC4 | pCfB4780 | pEYK, *Tm*XXT2, *Tm*CSLC4, Tlip | Expression of *Tm*XXT2+*Tm*CSLC4 | This study; Gibson Assembly |
| pCfB4780+*Tm*XXT2+*Tm*CSLC4+*Bd*XyBAT1 | pCfB4780 | pEYK, *Tm*XXT2, *Tm*CSLC4, *Bd*XyBAT1, Tlip | Expression of *Tm*XXT2+*Tm*CSLC4+*Bd*XyBAT1 | This study; Gibson Assembly |

Table S5. *Yarrowia lipolytica* strains used in this work.

| Strain | Genotype | Origin |
|---|---|---|
| Po1d | MatA, ura3-302, leu2-270, xpr2-322 | Barth, G & Gaillardin, C (1997) |
| $Yl^{AkCSLA3}$ | Po1d + pEYK_*Ak*CSLA3_Tlip + pTEF_Nat_Tcyc | This study |
| $Yl^{AkCSLA3+AkTBL25}$ | Po1d + pEYK_*Ak*CSLA3_Tlip + pEYK_*Ak*TBL25_Tlip + pTEF_Nat_Tcyc | This study |
| $Yl^{TmXXT2+TmCSLC4}$ | Po1d + pEYK_*Tm*XXT2_Tlip + pEYK_*Tm*CSLC4_Tlip + pTEF_Nat_Tcyc | This study |
| $Yl^{TmXXT2+TmCSLC4+BdXyBAT1}$ | Po1d + pEYK_*Tm*XXT2_Tlip + pEYK_*Tm*CSLC4_Tlip + pEYK_*Bd*XyBAT1_Tlip + pTEF_Nat_Tcyc | This study |
